# Machine Learning-Driven Transcriptome Analysis of Keratoconus for Predictive Biomarker Identification

**DOI:** 10.3390/biomedicines13051032

**Published:** 2025-04-24

**Authors:** Shao-Hsuan Chang, Lung-Kun Yeh, Kuo-Hsuan Hung, Yen-Jung Chiu, Chia-Hsun Hsieh, Chung-Pei Ma

**Affiliations:** 1 Department of Biomedical Engineering, Chang Gung University, Taoyuan 33302, Taiwan; 2 Department of Ophthalmology, Linkou Chang Gung Memorial Hospital, Taoyuan 33305, Taiwan; 3 College of Medicine, Chang Gung University, Taoyuan 33302, Taiwan; 4 Division of Oncology, Department of Internal Medicine, Linkou Chang Gung Memorial Hospital, Taoyuan 33305, Taiwan; 5 Department of Biomedical Sciences, College of Medicine, Chang Gung University, Taoyuan 33302, Taiwan

**Keywords:** keratoconus, machine learning, inflammation, transcriptome, biomarkers

## Abstract

**Background:** Keratoconus (KTCN) is a multifactorial disease characterized by progressive corneal degeneration. Recent studies suggest that a gene expression analysis of corneas may uncover potential novel biomarkers involved in corneal matrix remodeling. However, identifying reliable combinations of biomarkers that are linked to disease risk or progression remains a significant challenge. **Objective:** This study employed multiple machine learning algorithms to analyze the transcriptomes of keratoconus patients, identifying feature gene combinations and their functional associations, with the aim of enhancing the understanding of keratoconus pathogenesis. **Methods:** We analyzed the GSE77938 (PRJNA312169) dataset for differential gene expression (DGE) and performed gene set enrichment analysis (GSEA) using Kyoto Encyclopedia of Genes and Genomes (KEGG) pathways to identify enriched pathways in keratoconus (KTCN) versus controls. Machine learning algorithms were then used to analyze the gene sets, with SHapley Additive exPlanations (SHAP) applied to assess the contribution of key feature genes in the model’s predictions. Selected feature genes were further analyzed through Gene Ontology (GO) enrichment to explore their roles in biological processes and cellular functions. **Results:** Machine learning models, including XGBoost, Random Forest, Logistic Regression, and SVM, identified a set of important feature genes associated with keratoconus, with 15 notable genes appearing across multiple models, such as *IL1R1*, *JUN*, *CYBB*, *CXCR4*, *KRT13*, *KRT14*, *S100A8*, *S100A9*, and others. The under-expressed genes in KTCN were involved in the mechanical resistance of the epidermis (*KRT14*, *KRT15*) and in inflammation pathways (*S100A8/A9*, *IL1R1*, *CYBB*, *JUN*, and *CXCR4*), as compared to controls. The GO analysis highlighted that the *S100A8/A9* complex and its associated genes were primarily involved in biological processes related to the cytoskeleton organization, inflammation, and immune response. Furthermore, we expanded our analysis by incorporating additional datasets from PRJNA636666 and PRJNA1184491, thereby offering a broader representation of gene features and increasing the generalizability of our results across diverse cohorts. **Conclusions:** The differing gene sets identified by XGBoost and SVM may reflect distinct but complementary aspects of keratoconus pathophysiology. Meanwhile, XGBoost captured key immune and chemotactic regulators (e.g., *IL1R1*, *CXCR4*), suggesting upstream inflammatory signaling pathways. SVM highlighted structural and epithelial differentiation markers (e.g., *KRT14*, *S100A8/A9*), possibly reflecting downstream tissue remodeling and stress responses. Our findings provide a novel research platform for the evaluation of keratoconus using machine learning-based approaches, offering valuable insights into its pathogenesis and potential therapeutic targets.

## 1. Introduction

Keratoconus (KTCN) is a corneal ectatic disease characterized by local structural changes, reduced biomechanical strength, and thinning of the central cornea, which leads to protrusion and irregular astigmatism, ultimately causing vision loss [1]. It primarily affects the younger population, with a global prevalence of 1.38/1000 [2]. While the disease is prevalent worldwide, certain ethnic groups are more susceptible, especially those in the Middle East and South Asia, where higher incidence rates have been observed [3]. However, the pathogenesis of keratoconus remains under investigation. Due to insufficient understanding of the factors driving disease progression, the diagnosis, prevention, and management of the condition remain a significant challenge. Keratoconus is a complex multifactorial disease and has been reported to be associated with various etiologies, including environmental factors, oxidative stress, genetics, nutritional metabolism, comorbidities, and eye rubbing, as well as an increased expression of matrix metalloproteinases (MMPs) and pro-inflammatory mediators [4,5,6]. Pathologically, keratoconus is associated with distinct changes in the corneal microstructure, from the corneal epithelium, Bowman’s layer, to the stroma, including alterations in collagen fiber arrangement, density, diameter, and proteoglycan content, as well as a decrease in stromal cells, which leads to changes in macroscopic biomechanical properties [7,8]. Depending on the disease’s progression, changes in structure and extracellular matrix (ECM) components in keratoconus range from expansive alterations to tissue fibrosis. Additionally, fibrosis in the tissue suggests that the ECM may have undergone a repair and remodeling process, attempting to compensate for the degeneration caused by the disease [9]. However, specific biomarkers related to disease progression, the sequence of events, and the contribution of biomechanical factors remain undefined. Therefore, understanding the staged changes in the corneal microstructure of keratoconus, biochemical factors, or specific biomarkers involved in the process and the relationship of corneal tissues in disease development is crucial for diagnosis and treatment.

Recent studies on keratoconus suggest that its progression may involve the dysregulation of inflammatory responses and immune pathways [10,11,12]. Dou et al. used single-cell RNA sequencing (scRNA-seq) to analyze differentially expressed genes in corneal epithelial cells (CECs), corneal stromal cells (CSCs), and immune cells within the corneal stroma (ImCs), respectively [12]. They identified two potential novel biomarkers, *CTSD* and *CTSK*, in CSCs and examined their role in protein degradation, which could contribute to the imbalance and remodeling of collagen and ECM in keratoconus. The study also observed abnormal expression of the *SPRR1B* gene in CECs, suggesting that epithelial cell changes might be overlooked in clinical diagnosis. Moreover, in ImCs within the corneal stroma, increased expression of *IL23A* and *CXCL1* and decreased expression of the anti-inflammatory gene *IL1RN* further confirmed the important role of inflammation and immune regulation in keratoconus progression. The results also showed that ligand–receptor binding associated with protease inhibition (e.g., TIMP1-EGFR) and anti-inflammatory processes (e.g., ANXA1-FPR1 and IL1RN-IL1R1) was notably absent in various cells in keratoconus, providing deeper insights for diagnosis and treatment. Additionally, as genomic research indicates, proteolysis enzymes regulate collagen degradation and play an essential role in ECM reconstruction [12,13]. Imbalance in these factors can lead to tissue structural changes and the loss of biomechanical function. ECM remodeling is significantly influenced by various biochemical factors or biomarkers, including extracellular matrix components, cell mediators, inflammatory factors, hormones, metabolites, and chemical elements [14,15]. To understand the complexity of keratoconus pathogenesis, previous studies have examined changes in various biochemical factors or biomarkers using corneal tissues (epithelium and stroma), primary cultured corneal cells, tears, aqueous humor, and blood from keratoconus patients [16,17,18,19,20]. These studies suggest that changes in disease-associated factors may affect ECM remodeling dynamics, such as corneal collagen degradation or crosslinking, and contribute to the disease’s onset and progression. Therefore, identifying key biochemical factors or biomarkers associated with the disease will help in diagnosis, predicting or mitigating disease worsening, and developing targeted therapies to improve prognosis.

Corneal topography, while valuable, presents challenges in early diagnosis, often requiring the integration of multiple diagnostic parameters for comprehensive evaluation [21]. In contrast, genetic testing has emerged as a crucial tool in disease risk assessment. Variations or alterations in gene expression can indicate functional abnormalities within cells, and distinct gene expression patterns may be associated with disease onset, progression, and prognosis. Despite the potential of genetic testing, traditional single-marker biomarkers have limitations in clinical diagnosis, particularly for complex multifactorial diseases like keratoconus. Consequently, the focus has shifted toward identifying combinations of multiple biomarkers, which could provide more reliable insights into disease risk and progression. However, identifying relevant biomarker combinations remains a significant challenge. Further clinical trials are necessary to validate these associations and establish their diagnostic feasibility and clinical threshold accuracy.

In recent years, artificial intelligence and machine learning (AI/ML) have garnered significant attention and have been extensively applied in the field of biomedical engineering, particularly in areas such as drug development, biomedical imaging, and protein structure prediction. This widespread adoption can largely be attributed to the availability of large-scale datasets from open repositories in these domains [22,23,24]. Traditional statistical approaches typically assume that data follow a normal distribution; however, genomic datasets often do not conform to this assumption, rendering conventional parametric analyses, such as t-tests and ANOVA, unsuitable. In genomic studies, extreme values (such as gene mutations or genes with excessively high expression levels) or other characteristics deviating from normality can distort the data distribution. These extreme biomarkers may skew the distribution, complicating the data and limiting the effectiveness of traditional statistical methods in identifying such variations. As the scale of genomic datasets increases, both the dimensionality and complexity of the data expand, many of which exhibit nonlinear interrelationships. These high-dimensional datasets present significant challenges in visualization, further impeding the application of traditional statistical methods in genomic research [25]. As a result, scientists have increasingly turned to machine learning and deep learning approaches to address these challenges of nonlinear relationships and high-dimensional data visualization. In omics research, AI technologies have demonstrated their capacity to learn from diverse datasets and uncover underlying feature patterns, facilitating the identification of biomarkers linked to diseases. These models exhibit strong generalization capabilities and broad applicability. Studies have leveraged machine learning and deep learning techniques to discover novel biomarkers and predict drug efficacy, with findings indicating that these new biomarkers outperform traditional ones in predicting drug response [26]. The identification and quantification of these novel biomarkers offer substantial potential for advancing research and the development of innovative technologies. Current AI/ML-based predictions frequently use methods such as Logistic Regression, SVM, XGBoost, LightGBM, and Multilayer Perceptron (MLP) to mine high-risk factors or potential biomarkers related to disease risk and progression from large clinical datasets, such as transcriptome data [27,28,29]. These biomarkers have the potential to become important tools for early diagnosis and risk assessment. Therefore, AI-assisted methods not only improve diagnostic accuracy but also help identify appropriate intervention timing for more precise disease management [29].

The data for this study were sourced from the GEO database (GSE77938, Non-KTCN (control): 25, KTCN: 25). We used machine learning methods, including Logistic Regression, SVM, Random Forest, and XGBoost, to analyze the transcriptomes of keratoconus patients and normal corneas, focusing on disease pathways related to inflammation and metabolism (KEGG pathways). We further quantified the importance of feature genes in predicting keratoconus risk using SHAP (Shapley Additive Explanations) to identify highly relevant biomarker combinations. Clinical statistics typically rely on the relationship between genes and phenotypes, whereas machine learning considers not only the gene–phenotype relationship but also interactions and nonlinear relationships between features. AI technology has been widely used in research on topographic maps related to keratoconus progression [30,31]. However, AI screening models for keratoconus transcriptomes remain underdeveloped and underexplored. This study was the first to use machine learning to construct a risk prediction model for keratoconus and identify potential feature genes. We evaluated the model’s performance using metrics including sensitivity, specificity, accuracy, correlation coefficients, and receiver operating characteristic (ROC) curves with the area under the curve (AUC).

## 2. Materials and Methods

### 2.1. Dataset Collection

This study utilized the GSE77938 dataset from the Gene Expression Omnibus (GEO) database, which comprises 25 samples from keratoconus (KTCN) patients and 25 samples from control corneas. The datasets GSE77938 (PRJNA312169), PRJNA636666, and PRJNA1184491 were obtained and downloaded from the NCBI Bioproject, available at https://www.ncbi.nlm.nih.gov/bioproject/ (accessed on 1 September 2024). The RNA sequencing (RNA-Seq) data from datasets GSE77938 [32] were generated using an Illumina HiSeq 1500 platform (Illumina, San Diego, CA, USA) with paired-end sequencing. The transcriptomic samples were obtained from corneas and processed for total RNA extraction. For library preparation, RNA was processed using the TruSeq Stranded Total RNA Low Throughput with Ribo-Zero™ Human/Mouse/Rat Kit (Illumina, San Diego, CA, USA) according to the manufacturer’s protocol.

### 2.2. Data Processing and Identifying Differentially Expressed Genes (DEGs)

Differential gene expression (DGE) analysis was conducted to identify genes with significant expression differences between the two groups. The analysis was performed using the DESeq2 package (version 1.38.3, Bioconductor, Boston, MA, USA), with filtering criteria set to an adjusted *p*-value (padj) < 0.01 and a |log2FoldChange| > 2. These thresholds were applied to ensure that the selected genes exhibited substantial differential expression. To further process the data, quantile normalization and log transformation were applied to the raw counts to minimize systematic biases. DEGs were identified based on these criteria. For visualization of the DEGs, heatmaps and volcano plots were generated using the pheatmap, dplyr, ggplot2, and ggrepel packages in R 4.3.2, which facilitated the subsequent interpretation and analysis of the results.

### 2.3. Gene Function Enrichment Analysis

We employed gene set enrichment analysis (GSEA) and the g.profiler tool to investigate differences in gene expression between keratoconus and normal phenotypes. g.profiler is an advanced tool designed to identify gene sets linked to specific biological processes or diseases. We used g.profiler to perform gene set enrichment analysis, focusing on genes associated with Kyoto Encyclopedia of Genes and Genomes (KEGG) pathways. Through this step, we identified gene sets related to KEGG pathways that may be involved in the development of KTCN [33,34,35]. GSEA ranks genes based on their correlation with the phenotype and calculates an Enrichment Score (ES) for each gene set. A higher ES indicates a stronger association between the gene set and the phenotype. For this analysis, we selected the c2.cp.kegg.v11.0.symbols gene set from the Molecular Signatures Database (MSigDB), which contains genes linked to KEGG pathways. To improve the accuracy of the analysis, we applied a False Discovery Rate (FDR) threshold of <0.05. FDR is an important statistical measure that helps control the rate of false positives, ensuring a balance between sensitivity and specificity in the results, which is critical in gene set enrichment analysis [34]. Finally, we conducted GSEA using the R programming language to explore significantly enriched gene sets and their associations with keratoconus and normal corneas. Statistical significance was assessed with a *p*-value < 0.05, and enrichment curves were plotted to further validate the gene sets that were significantly enriched in the two phenotypes.

### 2.4. Protein–Protein Interaction (PPI) Network Construction and Gene Ontology (GO) Pathway Analysis

Genes across all machine learning models were selected and included in the PPI network analysis [36]. The PPI network was based on the STRING database (https://string-db.org/ (accessed on 10 January 2025)) with a confidence score threshold set to 0.5. The CytoHubba plugin in the Cytoscape software (version 3.8.0, Cytoscape Consortium, San Diego, CA, USA) was used to assess the importance of genes in the network, and genes with a degree ≥ 10 were considered hub genes. These selected genes were then subjected to Gene Ontology (GO) enrichment analysis to identify their roles in various biological processes (BPs), cellular components (CCs), and molecular functions (MFs). The statistical threshold was set to *p*-value < 0.05. A bar plot of the GO enrichment results was created using ggplot2.

### 2.5. Machine Learning Models

We employed four machine learning algorithms, including XGBoost (eXtreme Gradient Boosting), Random Forest, Logistic Regression, and Support Vector Machine (SVM). ML is a branch of AI that focuses on constructing models for prediction or recommendation systems, particularly well suited for analyzing complex relationships in large-scale data systems [37]. By analyzing intricate datasets, machine learning has become a powerful tool in enhancing the accuracy and speed of clinical research. It not only increases the translational value of data but also helps identify novel biomarkers and assess therapeutic efficacy.

XGBoost is an algorithm based on gradient boosting that has been optimized for better performance in gene expression data analysis, demonstrating strong predictive capabilities. It identifies key disease-related genes through feature importance analysis [38]. Beyond standard gradient boosting, XGBoost integrates L1 and L2 regularization to reduce overfitting and enhance generalizability. This model improves predictions by progressively constructing decision trees, where each new tree is designed to correct the residual errors of the previous ones, thereby progressively optimizing overall performance. The predicted value of each decision tree is multiplied by its corresponding weight, and the final prediction is obtained by summing the weighted predictions of all trees. The range of parameters for XGBoost that we used was as follows: colsample_bytree = 0.9, learning_rate = 0.1, max_depth = 10, and n_estimators = 50.

Random Forest operates by performing multiple random samplings of the training dataset to generate multiple decision trees, demonstrating a high tolerance to missing data. In gene data analysis, where there may exist nonlinear relationships or interactions between genes, Random Forest helps in the selection of feature genes associated with diseases [27]. Each decision tree in Random Forest is a part of the model, and, by combining multiple models, the prediction accuracy is enhanced. These predictions can be either categorical labels or continuous values. During the training process, random sampling of the original dataset ensures that each decision tree is trained on different data subsets, thus making the model more robust. The parameters for the Random Forest model that we used were as follows: max_depth = 10, min_samples_split = 5, and n_estimators = 100.

Logistic Regression is a commonly used binary classification linear model, often employed to predict the probability that a sample belongs to a specific class, explaining how each variable influences the prediction. It can predict the probability of disease occurrence based on gene expression or features [39]. The goal of logistic regression is to map the linear combination $\omega^{T}\chi_{i}+b$ to a probability between 0 and 1, thereby classifying the sample and determining which class it belongs to based on the predicted probability:

(1) $${\hat{Y}}_{i}=\frac{1}{1+e^{-{(\omega }^{T}\chi_{i}+b)}}$$

where ${\hat{Y}}_{i}$ is the predicted probability for the i-th sample, ω is the weight vector of the model containing the weights of all features, $\chi_{i}$ is the feature vector of the i-th sample, and b is the bias term representing the prediction when all features are zero. The training of the logistic regression model typically involves minimizing the loss function using gradient descent, with the objective of finding the optimal weights ω and bias *b* for accurate predictions on new samples. The parameters for the Logistic Regression model that we used included the following: C = 50, max_iter = 5000, solver = ‘liblinear’.

SVM is a powerful classification algorithm that aims to find the optimal hyperplane to separate data points of different classes. In gene expression data analysis, SVM can effectively classify diseases. SVM is widely applied in classification and regression analysis, with its operation based on maximizing the margin, hyperplanes, kernel tricks, and soft margins, which contribute to its excellent performance on complex datasets [24,27,28,40,41]. SVM is not limited to linear problems and can address nonlinear issues through kernel tricks, making it very useful for handling more complex datasets. The parameters for the SVM model that we used were as follows: kernel = ‘rbf’, C = 100, gamma = 0.01, and probability = True. During model training, we used 10-fold cross-validation (Stratified K-Fold Cross-Validation), which continuously monitors model performance and prevents overfitting, thus enhancing the model’s generalization ability. The dataset was divided into 10 subsets, with each subset containing a similar ratio of positive and negative samples, ensuring that the class distribution in each subset is consistent with the overall dataset. In each cross-validation iteration, the model was trained on the training set and evaluated on the test set:

(2) $${CV}_{fold}=\frac{1}{K}\sum_{k=1}^{K}Performance\;on\;{fold}_{k}$$

where $Performance\;on\;{fold}_{k}$ represents the performance metrics on the test set for the k-th fold. For each round of predictions, we calculated several performance metrics, including accuracy, sensitivity, specificity, precision, F1-score, Matthews Correlation Coefficient (MCC), and receiver operating characteristic (ROC) curve with area under the curve (AUC), to quantify the model’s performance at different decision thresholds [42,43,44].

### 2.6. SHAP Feature Selection

We further used the SHAP (SHapley Additive exPlanations) method to interpret the model’s predictions and compared the selected feature genes with the existing literature to validate the findings. Due to the intricate and often opaque internal structure of machine learning algorithms, determining the biological significance of selected features is frequently more challenging than conducting clinical statistical tests, which tend to provide more straightforward and interpretable results. SHAP method leverages cooperative game theory to attribute credit to the individual contributions of input features in machine learning algorithms [45,46]. It assigns a specific quantitative value to each feature and determines its predictive power. The formal mathematical representation of this is shown in Equation (3), which quantifies the incremental effect of adding a feature, denoted as *i*, to various subsets of features.

(3) $$\phi_{i}=\sum_{s\subseteq N\backslash \{i\}}\frac{|S|!\;(|N|-|S|-1)!}{|N|!}\;[f\;(S\cup \{i\})-f(S)]$$

where $\phi_{i}$ represents the SHAP value for the feature, N denotes the set of all features, S is a subset of features excluding i, f(S) is the model’s prediction based on the features in S, and f (S∪{i}) represents the prediction when feature i is included.

### 2.7. Statistical Analysis

This study employed R software 4.4.1 and Python 3.10.11 for data processing, statistical analysis, and result visualization. Statistical significance was defined as a *p*-value < 0.05 or *p*-value < 0.01 and was used to evaluate all comparisons and analyses. The data processing steps included imputation of missing values, data filtering, standardization, and transformation. In R, we used the DESeq2 package for differential gene expression analysis and the clusterProfiler, fgsea, ggplot2, and other packages for GSEA, analyzing significant gene sets related to KEGG pathways and visualizing the results. In Python, data analysis and feature selection were conducted using pandas, scikit-learn, and other packages, and machine learning models were developed. To better explain the model’s predictions, we used the SHAP package for interpretability analysis and visualized results with matplotlib and seaborn.

## 3. Results

**Figure 1:**
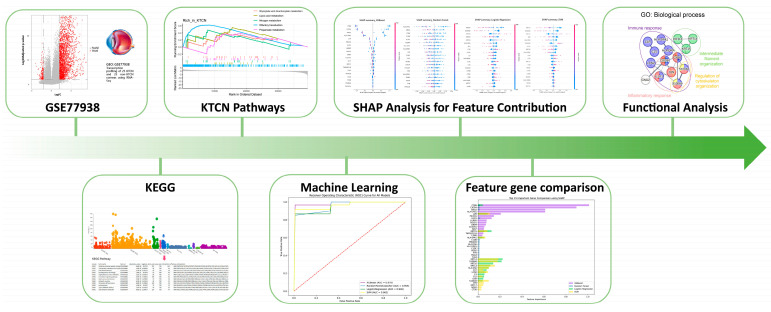
The workflow of this study.

**Figure 2:**
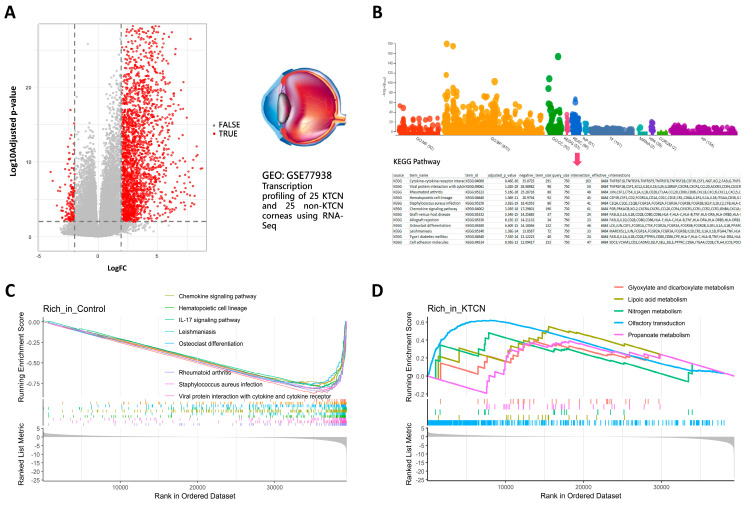
Identification of DEGs and GSEA results: (**A**) Volcano plot of differentially expressed genes. (**B**) Pathway annotation of DEGs using g.profiler. (**C**) The main signaling pathways significantly enriched in the control group based on GSEA. (**D**) The main signaling pathways significantly enriched in KTCN group based on GSEA.

The research framework is illustrated in Figure 1. Raw gene expression data for keratoconus were obtained from the GEO database. Following DGE analysis, a machine learning approach was employed to analyze the transcriptomic dataset and identify key feature genes. Subsequently, functionally related genes were examined. Figure 2 presents the transcriptomic dataset GSE77938, which included 25 control and 25 KTCN samples. DGE analysis identified 2429 significantly differentially expressed genes. To further explore biological pathways, KEGG pathway analysis was performed, followed by GSEA. The results revealed that metabolic pathways, such as nitrogen metabolism and lipoic acid metabolism, as well as olfactory transduction, a pathway involved in sensory signaling, were significantly enriched in KTCN. In contrast, immune and anti-inflammatory pathways, such as the chemokine signaling pathway, IL-17 signaling pathway, and pathways related to immune responses in hematopoietic cell lineage, Leishmaniasis, Staphylococcus aureus infection, and rheumatoid arthritis, were enriched in control. These findings suggested a distinct molecular landscape between KTCN and control, with metabolic pathways playing a predominant role in KTCN, while immune and inflammatory responses are more prominent in control. This differential enrichment highlights potential pathogenic mechanisms underlying keratoconus and provides insights for further functional investigations.

**Figure 3:**
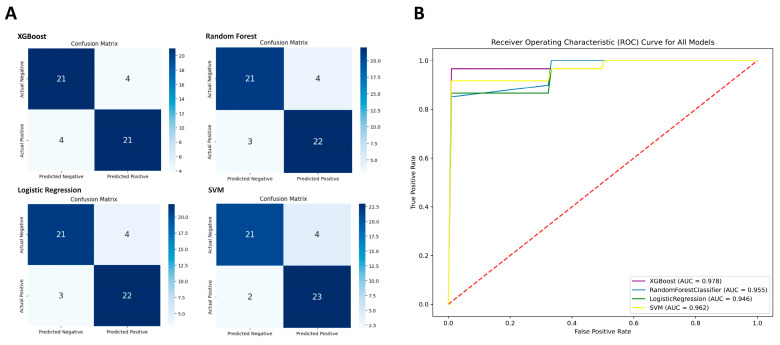
Comparative performance of machine learning models: (**A**) Confusion matrix visualizing classification outcomes. (**B**) ROC curves and AUC values illustrating model discriminative ability.

The performance of XGBoost, Random Forest, Logistic Regression, and SVM was evaluated based on multiple evaluation metrics (Table 1 and Figure 3). Additionally, the predicted performance of these models was visually interpreted through a confusion matrix (Figure 3A). The models were assessed based on True Positive (TP), True Negative (TN), False Positive (FP), False Negative (FN), Sensitivity, Specificity, Accuracy, F1-score, the MCC, and the AUC. The XGBoost model achieved 84% sensitivity, 84% specificity, and 84% accuracy, with an F1-score of 0.84 and a MCC of 0.86, reflecting its strong performance. Its ROC-AUC was 0.97 (Figure 3B), indicating excellent discriminative ability. Random Forest showed similar sensitivity and specificity but had a slightly lower ROC-AUC of 0.95. Logistic Regression demonstrated lower discriminative power compared to the other models. SVM outperformed all models, with the highest accuracy (88%), F1-score (0.885), and sensitivity (92%), although its specificity remained at 84%. The MCC for SVM was 0.76, and the ROC-AUC was 0.96, reflecting strong overall performance.

Based on model performance, SVM was the most effective for predicting KTCN in the GSE77938 dataset, achieving an MCC of 0.76, indicating a strong correlation between predicted and actual values and a good balance in detecting both KTCN and control samples. XGBoost, despite having lower sensitivity, demonstrated a superior ROC-AUC and a higher MCC, highlighting its strong discriminative ability. For scenarios prioritizing the accurate detection of positive samples, SVM may be the better choice due to its high sensitivity and F1-score. However, XGBoost can better distinguish between KTCN and control groups in such situations, especially when prediction accuracy depends on the clear separation of the two classes, due to its higher ROC-AUC and MCC.

**Figure 4:**
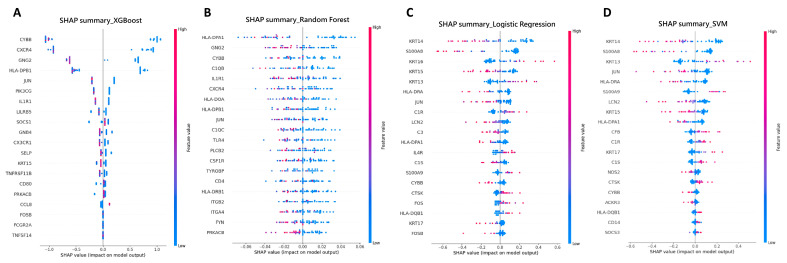
SHAP feature contribution analysis for (**A**) XGBoost, (**B**) Random Forest, (**C**) Logistic Regression, (**D**) SVM. Each subplot visualizes SHAP values, indicating the contribution of selected features to model predictions.

Furthermore, we employed SHAP analysis to interpret the predictions made by each machine learning model and assess the contribution of individual feature genes to those predictions. SHAP allowed us to assess the importance of each feature gene, providing insights into how these genes influenced the likelihood of KTCN versus non-KTCN outcomes. By using SHAP in conjunction with machine learning models, we enhanced the interpretability of the predictions. SHAP provide insight into the influence of each feature on the model’s predictions, thereby allowing for the more accurate identification of genes that are strongly associated with the disease. Figure 4 displays the main features identified for this study. Each row in the charts represented a different feature, with each dot corresponding to the SHAP value for that feature in a specific sample. Red dots indicated a high feature value, while blue dots represent a low feature value. The horizontal axis showed the SHAP values, which quantified the impact of each feature on the model’s predictions. In our study, we used SHAP to interpret the predictions of the XGBoost model and identified feature genes primarily related to immune function. In contrast, although SVM performed well in distinguishing between disease and control groups, its ability to handle complex issues involving multiple gene interactions was limited. Despite this, our analysis identified notable correlations between the reduced expression of *S100A8*, *KRT14*, and *KRT15* and the increased expression of *S100A9* and *KRT13*, all of which were strongly linked to an increased risk of keratoconus.

**Figure 5:**
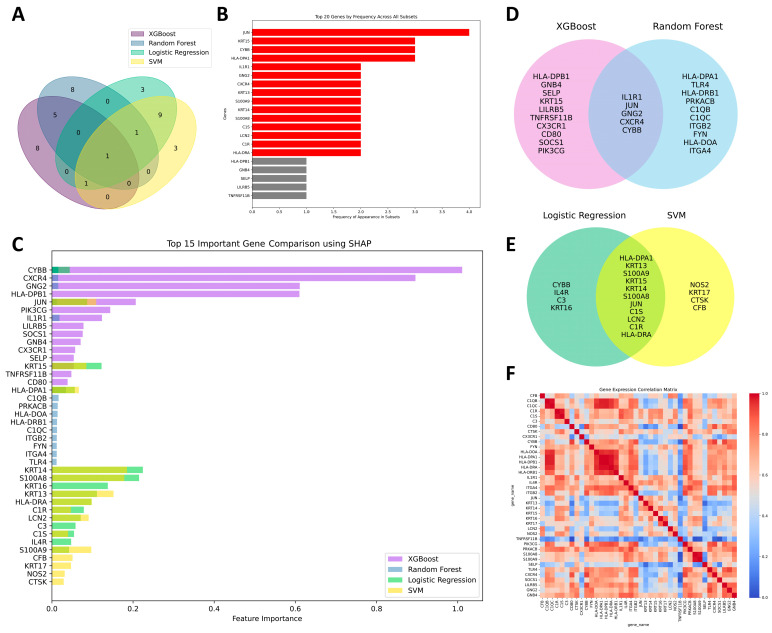
Summary of 60 feature genes selected by machine learning models: (**A**) Overlap of selected features among the four models (diagram showing intersection counts without gene names). (**B**) Top 20 most frequently selected genes across all subsets. (**C**) Overlapping genes across the four models. (**D**) Shared genes between XGBoost and Random Forest. (**E**) Shared genes between Logistic Regression and SVM. (**F**) Pearson correlation matrix of the feature genes selected by machine learning models shown in the heatmap.

A total of 60 genes selected by machine learning models, with each model identifying its top 15 feature genes (Figure 5). Figure 5A presents the overall overlap of selected genes among the four models, displaying intersection counts without gene names. The top 20 most frequently selected features across all model subsets are highlighted as shown in Figure 5B. *JUN* was the only gene that appeared in all model subsets, followed by *KRT15*, *CYBB*, and *HLA-DPA1*. Additionally, *IL1R1*, *GNG2*, *CXCR4*, *KRT13*, *KRT14*, *S100A8*, *S100A9*, *C1S*, *LCN2*, *C1R*, and *HLA-DRA* were present in at least two of the four model subsets. We further examined which machine learning models selected each feature gene and assessed the degree of similarity in gene selection among the models. We compared the overlap of selected feature genes across models and visualized their intersections (Figure 5C). To investigate the relationships between specific models, we focused on the genes commonly selected by XGBoost and Random Forest and the shared genes between Logistic Regression and SVM (Figure 5D,E). These visualizations provide insights into the consistency of feature selection across different machine learning models. *IL1R1*, *JUN*, *GNG2*, *CXCR4*, and *CYBB* were identified as common genes for XGBoost and Random Forest, while *HLA-DPA1*, *KRT13*, *KRT14*, *KRT15*, *S100A8*, *S100A9*, *JUN*, *C1S*, *LCN2*, *C1R*, and *HLA-DRA* were shared between Logistic Regression and SVM. We further performed correlation analysis on the datasets after feature selection by the four ML models. The heatmap represents the Pearson correlation matrix of the 40 feature genes selected by the machine learning models (Figure 5F). These findings suggested that XGBoost and Random Forest shared similarities in their feature importance evaluation methods because they both are decision tree-based models. Although SVM employed a nonlinear radial basis function (RBF) kernel in our study, both Logistic Regression and SVM exhibited greater overlap in feature selection due to their shared tendency to select features that contribute significantly to the decision boundaries, particularly when the data revealed clearer patterns that may not require highly complex nonlinear modeling. Overall, these results highlight the importance of model choice in feature selection, as well as the relationship between the data characteristics and the ability of each model to capture relevant patterns.

**Figure 6:**
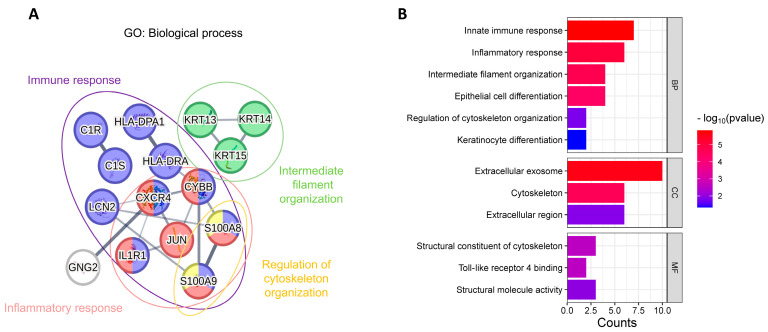
Functional analysis of top 15 selected genes across all machine learning models: (**A**) functional relationships among genes and (**B**) Gene Ontology (GO) functional enrichment analysis.

To explore the functional roles of these genes, GO enrichment analysis was performed, categorizing the genes into distinct BPs, CCs, and MFs. Figure 6A illustrates the top 15 feature genes across all machine learning models and their interaction relationships. The gene nodes represent genes within the network, with the color of each node indicating the biological processes they were involved in, while the thickness of the connecting lines reflect the strength of their interactions. The GO analysis highlighted that the *S100A8/A9* complex and its associated genes were primarily involved in biological processes related to the cytoskeleton organization, inflammation, and immune response. Furthermore, the molecular functions of toll-like receptor 4 binding and the structural constituent of the cytoskeleton are predominantly linked to the gene *S100A8/A9* complex, *KRT13*, *KRT14*, and *KRT15* (Figure 6B). These findings suggested potential mechanisms linking cytoskeleton organization and immune response, offering insights into their roles in regulating inflammation and tissue remodeling during disease progression.

**Figure 7:**
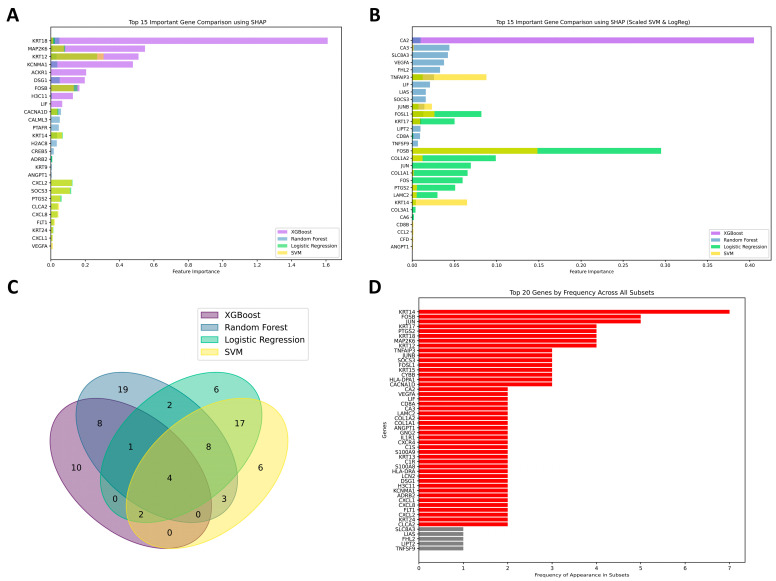
Overlap of genes across different datasets: (**A**) Genes from the PRJNA636666 dataset selected by machine learning models. (**B**) Genes from the PRJNA1184491 dataset selected by machine learning models. (**C**) Intersection of gene sets across the three datasets (PRJNA312169, PRJNA636666, and PRJNA1184491). (**D**) Top 50 most frequently selected features across all three datasets.

While the primary focus of our study was based on the GSE77938 (PRJNA312169) dataset, we further expanded our analysis by incorporating two additional, independent datasets (PRJNA636666 and PRJNA1184491) to increase the robustness and generalizability of our analysis. The details regarding these datasets are presented in Supplementary Table S1. The inclusion of these datasets aimed to capture a broader range of biological variability and further validate the significance of the identified genes, thereby providing a more comprehensive representation of gene features across different cohorts. Figure 7 presents the most significant genes identified across the different datasets (PRJNA312169, PRJNA636666, and PRJNA1184491) using various machine learning algorithms, highlighting their relevance and importance. Figure 7A,B, and Supplementary Figure S1 show the overlap of selected feature genes across models, with their intersections visualized separately for the PRJNA636666 and PRJNA1184491 datasets. Figure 7C presents the overall gene overlap across the three combined datasets. The top 50 most frequently selected features, identified after merging the three datasets, are highlighted in Figure 7D.

This analysis revealed that several genes exhibited significant overlap across the datasets. *KRT14* emerged as the only gene identified seven times, underscoring its pivotal role in maintaining cellular integrity and epithelial function. Specifically, *KRT14* is crucial for the structural stability and strength of the corneal matrix. In KTCN, a decrease in structural integrity may be linked to altered *KRT14* expression [47], positioning it as a potential target for future therapeutic strategies. Additionally, genes such as *FOSB* and *JUN*, which were identified five times, along with *KRT18*, *MAP2K6*, *KRT12*, *KRT17*, and *PTGS2* appearing four times, demonstrated consistent representation across datasets. The genes identified three times, including *CYBB*, *TNFAIP3*, *SOCS3*, *JUNB*, *CACNA1D*, *FOSL1*, *HLA-DPA1*, and *KRT15*, were frequently selected in machine learning feature analysis, highlighting their significant roles in immune response mechanisms, intercellular signaling, and structural maintenance of intermediate filaments. Furthermore, several genes were identified twice, such as *CA2*, *VEGFA*, *LIF*, *CD8A*, *CA3*, *LAMC2*, *COL1A2*, *COL1A1*, *ANGPT1*, *GNG2*, *IL1R1*, *CXCR4*, *C1S*, *S100A8*, *S100A9*, *KRT13*, *DSG1*, *C1R*, *HLA-DRA*, *LCN2*, *H3C11*, *KCNMA1*, *ADRB2*, *CXCL2*, *CXCL8*, *CXCA2*, *FLT1*, *KRT24*, and *CXCL1*. Their co-occurrence pointed to the complexity and the interconnectedness of multiple molecular processes.

**Figure 8:**
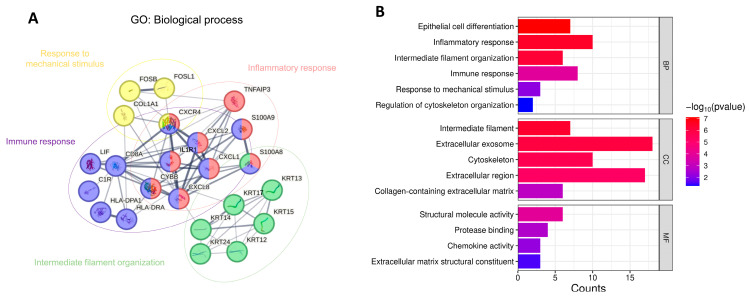
Functional analysis of top 45 feature genes shared across the three datasets: (**A**) functional relationships among genes and (**B**) Gene Ontology (GO) functional enrichment analysis.

Figure 8 demonstrates the GO analysis for the 45 feature genes shared across the three datasets, revealing their involvement in key biological processes such as cell differentiation, immune response, intermediate filament organization, response to mechanical stimulus, and cytoskeleton organization. These functions are critical for maintaining tissue structure, cellular communication, and adapting to external stress. Notably, *S100A8* overlapped across inflammatory response, intermediate filament organization, and immune response, indicating its dual role in structural maintenance and immune modulation. Likewise, *CXCR4* was enriched in response to mechanical stimulus, inflammatory response, and immune response, suggesting its capacity to integrate mechanical and immunological signals. In terms of molecular function, these genes were primarily associated with structural molecule activity, protease binding, and extracellular matrix components, further supported by cellular component enrichment pointing to the cytoskeleton and extracellular matrix. Collectively, these findings underscored the functional versatility of the shared genes and their relevance in both structural integrity and inflammatory regulation.

## 4. Discussion

According to the results of transcriptome analysis, 2428 significantly differentially expressed genes were identified in KTCN compared to the control group. Following KEGG pathway analysis, we further selected 211 genes through GSEA. Subsequently, several machine learning methods were employed along with SHAP analysis, leading to the identification of 15 shared feature genes: *JUN*, *CYBB*, *HLA-DPA1*, *IL1R1*, *GNG2*, *CXCR4*, *KRT13*, *KRT14*, *KRT15*, *S100A8*, *S100A9*, *C1S*, *LCN2*, *C1R*, and *HLA-DRA*. Functional analysis revealed that the *S100A8/A9* complex and its associated genes were primarily involved in biological processes related to cytoskeleton organization, inflammation, and immune response. The *S100A8/A9* complex (S100 family) is a key regulator of inflammatory responses and ECM remodeling, including the regulation of matrix metalloproteinases (MMPs) and glycosaminoglycans (GAGs) [10,48]. It has been shown to be associated with several MMPs involved in the degradation of collagen and GAGs [49]. Additionally, *KRT13*, *KRT14*, and *KRT15*, members of the keratin family, primarily provide structural support and mechanical strength to epithelial cells and are classified as cytoskeletal proteins [50]. The genes primarily involved in immune and inflammatory responses included *IL1R1*, *CYBB*, and *CXCR4*. Studies have reported that the loss of IL1RN-IL1R1 ligand–receptor binding may significantly impact the progression of keratoconus [12].

Traditionally, KTCN has been considered a non-inflammatory disease, with its pathogenesis primarily associated with elevated levels of degradative enzymes in corneal stroma, including gelatinase, MMPs, and peroxidases [51]. These enzymes are thought to contribute to the degradation of collagen [52]. However, these findings have mainly been confirmed through histological or biochemical examinations, which represent only partial pathological changes. In recent years, numerous studies conducted comprehensive analyses of the genomics, transcriptomics, and proteomics of keratoconus samples, uncovering more nuanced and precise pathogenic mechanisms, particularly through cell-type-specific investigations [10,19,32,53]. Dou et al. utilized single-cell RNA sequencing technology to identify two potential novel biomarkers, *CTSD* and *CTSK*, in corneal stromal cells [12]. These genes, which are involved in protein degradation, may play a significant role in the disruption of collagen and ECM remodeling in keratoconus. Additionally, the immune cells in the corneal stroma of KTCN exhibited a reduced expression of the anti-inflammatory gene *IL1RN*, along with the loss of ligand–receptor binding associated with IL1RN-IL1R1. In our study, the SVM model identified *CTSK*, although it did not overlap with genes identified by other ML approaches. Conversely, both XGBoost and Random Forest models identified *IL1R1*. Moreover, Sun et al. reported a significant association between immune–inflammatory genes and the pathogenesis pathways in their analysis of the keratoconus transcriptome [10]. Their study integrated corneal epithelium and blood samples from seven KTCN patients with transcriptomic datasets GSE77938 and GSE112155 for GO enrichment analysis. The analysis revealed the significant enrichment of genes such as *HLA-DQB1*, *HLA-DPA1*, *S100A8*, *SFRP1*, *CD247*, *CTSH*, and *LAMP3*, which are linked to inflammatory and immune disease pathways. Notably, *HLA-DPA1* and *S100A8* overlap with genes identified in our ML models. Furthermore, in our analysis, pathways such as chemokine signaling and IL-17 signaling were enriched in the control group. Several of these are associated with biological processes including hematopoietic cell lineage and rheumatoid arthritis consistent with the results reported by Sun et al. [10]. Proteomic studies by Lema and Balasubramanian in KTCN tear samples further highlighted the involvement of immune modulation in the disease progression, primarily marked by a reduction in immunoglobulin kappa chains and polymeric immunoglobulin receptors [54,55]. In addition, Nielsen et al. reported abnormal expressions of the MHC II alpha chain encoded by the *HLA-DRA1* gene in keratoconus [56]. Recent findings from East Asian populations have also identified associations between KTCN and pathways related to rheumatoid arthritis, highlighting the activation of immune cells and the release of inflammatory cytokines such as TNF-α, IL-1, and IL-6 [11]. These results reinforce the notion that the development of keratoconus may involve the dysregulation of inflammatory responses and immune regulatory pathways.

In our functional analysis, pathways associated with biological processes such as cytoskeleton organization and epithelial cell differentiation were found to be enriched, suggesting that the reorganization of the cytoskeleton also played a pivotal role in the pathogenesis of KTCN. The remodeling of the cytoskeleton, including dynamic alterations in microfilaments, microtubules, and intermediate filaments, directly impacts the interaction between cells and the ECM [57]. Through these dynamic interactions with the ECM, the cytoskeleton can sense and respond to external mechanical forces, thereby influencing cellular behavior. Therefore, the interaction between the cytoskeleton and the ECM is crucial for cellular mechanical properties. Our data revealed that the increased risk of KTCN was associated with the decreased expression of *S100A8*, *KRT14*, and *KRT15*, as well as the increased expression of *S100A9* and *KRT13*. These genes were likely crucial for maintaining corneal structural stability and mechanical integrity, particularly in the development and mechanical structure of the corneal epithelium and the ECM [58]. Previous studies also identified several genes known to be involved in KTCN, such as the downregulation of *KRT14* [47] and *S100A8* [50] in the corneal stroma and the upregulation of KRT16 protein [59], which were consistent with our findings from the ML-based gene selection. The dataset adopted in this study (GSE77938) was established by Kabza et al. [32], who reported pathways associated with KTCN, highlighting the regulation of Hippo, Wnt, and TGF-β pathways through the core YAP/TAZ. YAP/TAZ are key factors in the cellular response to mechanical stress, influencing cytoskeleton reorganization and cell behavior by regulating gene expression [12]. Our study further confirmed that feature genes such as the *S100A8/A9* complex, *KRT14*, and *KRT15* were significantly associated with the increased risk of keratoconus, and alterations in these genes were closely related to corneal structural stability, mechanical strength, and disease progression. These genes may also have been linked to the pathological mechanisms of keratoconus reported by Kabza et al., although Kabza’s focus was primarily on the regulation of signaling pathways. Our research extended Kabza et al.’s work with a different approach to gene analysis by applying machine learning in feature gene selection.

In our ML-based prediction analysis for dataset GSE77938, XGBoost and Random Forest jointly prioritized genes such as *IL1R1*, *JUN*, *GNG2*, *CXCR4*, and *CYBB*, predominantly associated with immune response and inflammation pathways. Meanwhile, SVM and Logistic Regression identified a broader panel including *HLA-DPA1*, *KRT13*, *KRT14*, *KRT15*, *S100A8*, *S100A9*, *JUN*, *C1S*, *LCN2*, *C1R*, and *HLA-DRA,* encompassing not only immune-related genes but also those involved in cytoskeleton organization and intermediate filament structure. XGBoost, a gradient boosting algorithm, improves model performance by iteratively minimizing prediction error, with built-in cross-validation to reduce overfitting [60]. Our SVM model, based on a nonlinear RBF kernel, showed notable overlap in feature selection with Logistic Regression, suggesting that data-inherent feature importance may dominate model-specific biases. Both SVM and Logistic Regression are supervised learning models aiming to maximize classification accuracy, often selecting features that directly impact the decision boundary. The gene sets selected by XGBoost and SVM may point to complementary dimensions of keratoconus pathophysiology. XGBoost captured upstream immune and chemotactic signals (e.g., *IL1R1*, *CXCR4*), whereas SVM highlighted epithelial structural and inflammatory markers (e.g., *KRT14*, *S100A8/A9*), potentially reflecting downstream remodeling and stress responses [61]. These findings supported the use of multiple ML models to achieve a more holistic molecular characterization of keratoconus, integrating both systemic and tissue-level mechanisms.

Our study primarily focused on evaluating the reliability of the ML model’s gene identification process, specifically for identifying potential biomarkers for progressive KTCN using dataset GSE77938 (PRJNA312169). By incorporating additional datasets from PRJNA636666 and PRJNA1184491, we were able to assess gene expression across multiple cohorts, offering a more comprehensive view of potential biomarkers. In contrast, Cheng et al. employed the GSE77938 and GSE151631 datasets to develop a predictive model based on oxidative stress (OS) and ECM remodeling genes [62]. They identified 454 DEGs and selected 17 oxidative stress and ECM-related DEGs (OEDEGs), which were validated using ML models. This study provided an in-depth look at the molecular signature of KTCN, with a particular focus on immune cell infiltration and the role of oxidative stress. Although similar datasets were used, our study advanced the methodology by incorporating several ML techniques for more refined gene selection. By applying the SHAP method, we assessed the contribution of each gene to predict KTCN, offering an interpretable approach for understanding how each feature gene influences the disease.

Additionally, Ouyang et al. utilized the same datasets to identify DEGs and long non-coding RNAs (lncRNAs) involved in KTCN [63]. They identified 20 key genes through protein–protein interaction (PPI) networks and proposed several miRNAs (e.g., *miR-4298* and *miR-4494*) as potential regulatory players in KTCN. While this study focused on non-coding RNAs, our research identified key protein-coding genes (e.g., *KRT14*, *JUN*, *KRT15*, *S100A8*, *COL1A1*) and extended the analysis by using machine learning to identify the most relevant genes across datasets. By employing multiple ML algorithms, we selected genes that showed consistent results across three datasets, offering a more comprehensive and reliable set of potential biomarkers for KTCN.

While previous studies have significantly advanced our understanding of keratoconus, our research introduces a novel approach by combining ML-driven gene selection with multi-dataset validation. This strategy enabled us to identify key genes across various cohorts, shedding light on their roles in immune regulation, cytoskeletal organization, and inflammation. Notably, *S100A8* was found to overlap across inflammatory response, intermediate filament organization, and immune response, suggesting its multifunctional role in modulating both structural and immunological processes. Similarly, *CXCR4* showed involvement in response to mechanical stimulus, inflammatory response, and immune response, highlighting its potential as an integrator of mechanical and immune signaling. The functional overlap of these genes across multiple GO terms emphasized their importance in orchestrating complex cellular behaviors and their relevance as potential therapeutic or diagnostic targets in inflammation-related conditions. The consistency of these genes across different datasets strengthened their potential as reliable biomarkers, emphasizing their relevance to the fundamental biological processes underlying KTCN. The use of multiple datasets further supports the credibility of our ML-based approach, confirming its ability to identify biomarkers with broad applicability in KTCN research. Our results suggested that, in KTCN tissue, several pathways were working together to protect the cornea and epithelium from inflammation and mechanical injury. However, further functional investigations will be required to determine whether this impairment is directly caused by defects in the KTCN stroma or is indirectly driven by other factors, such as the epithelium, tears, or immune cells. Understanding these underlying mechanisms will be crucial for identifying potential therapeutic and diagnostic targets, as well as developing more effective treatment strategies for KTCN.

### Limitation

In this study, we focused on the analysis of the GSE77938 dataset, with the primary objective of evaluating the feasibility of applying machine learning methods. It will be essential to integrate more transcriptomic data for the optimization of the AI model. By combining large-scale gene expression data, we aimed to investigate the relationship between the expression of feature genes and the progression of keratoconus. However, several factors, including RNA analysis methods, sequencing platforms, fragment lengths, sequencing depths, and gene expression normalization techniques used across different datasets, could introduce inconsistencies in feature genes. Moreover, the selection of datasets, features, factor weights, and machine learning classification algorithms may significantly affect the model’s accuracy, relevance, and generalizability. Additional variables such as sample size, gender, and ethnicity in the pooled datasets could impose further limitations. As such, the results may be specific to the dataset analyzed and may not be broadly applicable to other datasets or target populations.

To address these limitations and expand the scope of our findings, we incorporated additional datasets to improve the generalizability of our results. In our study, corneal samples from individuals with progressive KTCN were analyzed, as they are most relevant for identifying biomarkers associated with the disease. After applying ML for feature selection, several genes were consistently identified across different datasets, highlighting their importance in KTCN development. However, regarding mild KTCN cases, as well as other sample sources like epithelial tissue or blood, we did not observe a consistent set of genes that could be reliably used for ML analysis. This limitation was primarily due to the small sample size and insufficient differentially expressed genes identified through DEG analysis. Therefore, these sample types may not be suitable for inclusion in the ML analysis based on the current available data.

For future clinical applications, ML models could be applied to more specific and larger cohorts, particularly those including mild KTCN patients or samples from different tissue types like the corneal epithelium, tears, or blood. This would allow for a more comprehensive understanding of the biomarkers and improve the clinical detection accuracy of keratoconus. Future work would include collecting more diverse and larger datasets, especially focusing on earlier-stage KTCN, to further enhance the generalizability and precision of predictive models.

## 5. Conclusions

Based on the findings from this study, we developed a machine learning-based framework to identify gene combinations and their functional associations in keratoconus. By analyzing the GSE77938 dataset and utilizing multiple machine learning models, we identified several feature genes, such as *IL1R1*, *JUN*, *CYBB*, *CXCR4*, *KRT13*, *KRT14*, *KRT15, S100A8*, and *S100A9*, that are involved in processes related to cytoskeleton remodeling, mechanical resistance, and inflammation. Notably, the SVM model showed superior performance in terms of sensitivity and accuracy, while XGBoost demonstrated enhanced discriminative ability. The GO analysis highlighted the role of the *S100A8/A9* complex in regulating the cytoskeleton, immune response, and inflammation. Furthermore, two additional datasets were incorporated to improve the generalizability of the results, with the co-occurrence of several genes across the three datasets providing a broader representation of gene features across diverse cohorts. These findings support the integration of multiple machine learning models to achieve a more comprehensive molecular characterization of keratoconus, offering deeper insights into its pathogenesis and uncovering potential biomarkers and therapeutic targets. This study also underscores the importance of selecting appropriate machine learning models to improve predictions and risk assessments in complex diseases such as keratoconus.

## Figures and Tables

**Table 1:** Performance evaluation of machine learning models.

	XGBoost	Random Forest	Logistic Regression	SVM
**True Positive**	21	22	22	23
**True Negative**	21	21	21	21
**False Positive**	4	4	4	4
**False Negative**	4	3	3	2
**Sensitivity**	84%	88%	88%	92%
**Specificity**	84%	84%	84%	84%
**Accuracy**	84%	86%	86%	88%
**F1-score**	0.84	0.863	0.863	0.885
**MCC**	0.86	0.72	0.72	0.76
**AUC**	0.97	0.95	0.94	0.96

MCC: Matthews Correlation Coefficient. AUC: area under the curve.

## Data Availability

All the raw and RNA-seq files in this research were obtained from the GEO database. The code and processed data used for analysis are available from the corresponding author upon reasonable request.

## Supplementary Materials

The following supporting information can be downloaded at: https://www.mdpi.com/article/10.3390/biomedicines13051032/s1, Figure S1: Overlap of genes across different datasets; Table S1: Dataset information.

## Abbreviations

The following abbreviations are used in this manuscript:

KTCN	Keratoconus
DEGs	Differentially expressed genes
DGE	Differential gene expression
CECs	Corneal epithelial cells
CSCs	Corneal stromal cells
ImCs	Immune cells
GSEA	Gene set enrichment analysis
KEGG	Kyoto Encyclopedia of Genes and Genomes
GO	Gene Ontology
BPs	Biological processes
CCs	Cellular components
MFs	Molecular functions
SHAP	SHapley Additive exPlanations
MMPs	Matrix metalloproteinases
ECM	Extracellular matrix
RNA-Seq	RNA sequencing
scRNA-seq	Single-cell RNA sequencing
ROC Curves	Receiver operating characteristic curves
AUC	Area under the curve
AI	Artificial Intelligence
ML	Machine learning
MSigDB	Molecular Signatures Database
FDR	False Discovery Rate
GEO	Gene Expression Omnibus
TP	True Positive
TN	True Negative
FP	False Positive
FN	False Negative
MCC	Matthews Correlation Coefficient
GAGs	Glycosaminoglycans
SVM	Support Vector Machine
XGBoost	eXtreme Gradient Boosting
RBF	Radial basis function
