# Supplementary material for: Machine Learning-Driven Transcriptome Analysis of Keratoconus for Predictive Biomarker Identification
Source: Biomedicines. 2025 Apr 24;13(5):1032. doi: 10.3390/biomedicines13051032 (PMC12109562; doi:10.3390/biomedicines13051032)

Supplementary Table S1. Dataset information

GEO Accession	BioProject	Sample #	Source	Severity	Submission date	Reference/source
GSE77938	PRJNA312169	KTCN:25<br>Control:25	Medical University of Warsaw	Cornea tissue: severe	2016	Kabza M et al. [32]
GSE151631	PRJNA636666	KTCN:19<br>Control:7	Wilmer Eye Institute and King Khaled Eye Specialist Hospital	Cornea tissue: severe	2020	Shinde V et al. [11]
-	PRJNA1184491	KTCN:20<br>Control:16	Federal Scientific and Clinical Center for Physico-Chemical Medicine named after Yu.M. Lopukhin	Cornea tissue: advanced to severe	2024	FNCPCM (https://rcpcm.ru/)

Supplementary Figure S1. Overlap of genes across different datasets. (**A**) Genes and (**B**) top 20 most frequently selected genes from dataset PRJNA636666. (**C**) Genes and (**D**) top 20 most frequently selected genes from dataset PRJNA1184491.

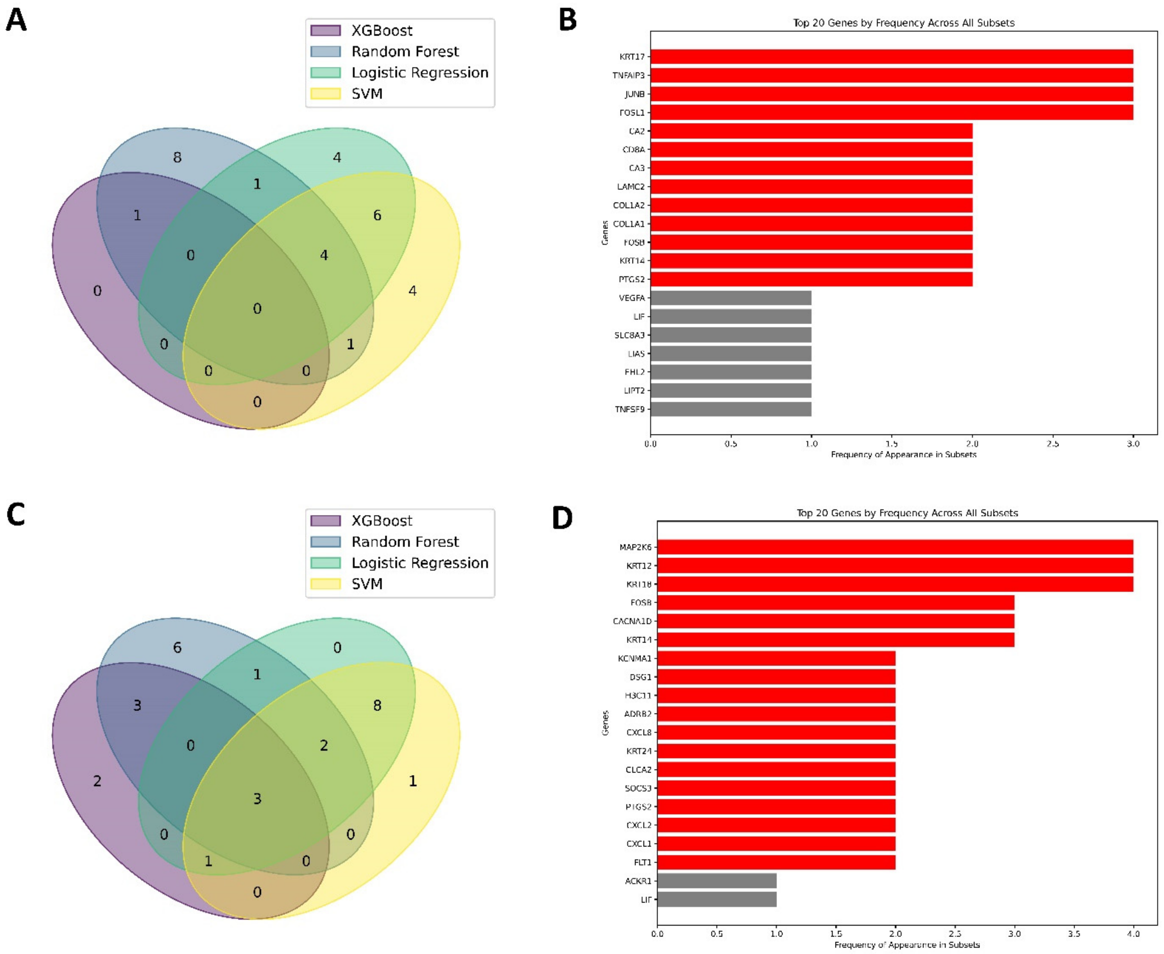

Supplement: Supplementary file 1 [file biomedicines-13-01032-s001.zip › biomedicines-3511118-supplementary.pdf]
